# Association and prediction of subjective sleep quality and postoperative delirium during major non-cardiac surgery: a prospective observational study

**DOI:** 10.1186/s12871-023-02267-x

**Published:** 2023-09-11

**Authors:** Jinwei Zheng, Lulu Wang, Wei Wang, Huafeng Zhang, Fangfang Yao, Junping Chen, Qingxiu Wang

**Affiliations:** 1 Department of anesthesiology, Shanghai East Hospital, School of Medicine, Tongji University, Shanghai, 200120 China; 2 Department of anesthesiology, Ningbo No.2 Hospital, Ningbo, 315000 Zhejiang China; 3 Nursing department, Ningbo No.2 Hospital, Ningbo, 315000 Zhejiang China

**Keywords:** Subjective sleep quality, Elderly, Major surgery, Postoperative delirium

## Abstract

**Background:**

Postoperative delirium (POD) is an acute form of brain dysfunction that can result in serious adverse consequences. There has been a link between cognitive dysfunction and poor sleep. The present study aimed to determine the association and prediction of subjective sleep quality and postoperative delirium during major non-cardiac surgery.

**Methods:**

One hundred and thirty-four patients, aged 60 years or older, were scheduled for elective laparotomy or orthopaedic procedures. The Pittsburgh Sleep Quality Index (PSQI) and sleep log were used to assess perioperative subjective sleep quality in participants. Nursing Delirium Screening Checklist (NU-DESC) was used for screening, and the Confusion Assessment Method (CAM) was used to diagnose POD during the first seven days following surgery. The association between subjective sleep quality and POD was assessed using a multivariate logistic regression model. Thereafter, the prediction performance of subjective sleep quality was evaluated using a receiver operating characteristic (ROC) curve.

**Results:**

All assessments were completed on 119 patients who had an average PSQI score of 7.0 ± 2.4 before surgery. 23 patients (19.3%) suffered from POD. The multivariate logistic regression analysis showed that the occurrence of POD was closely related to age, BMI, PSQI and operation time. After adjusting for related factors, there was a statistically significant association between PSQI and POD occurrence (OR = 1.422, 95%CI 1.079–1.873, per 1-point increase in PSQI). The ROC curve analysis showed that the optimal PSQI cutoff value was 8.0 for predicting POD, and the area under the ROC (AUROC) value of PSQI was 0.741 (95%CI 0.635 to 0.817). The AUROC of the model developed by the multivariate logistic regression analysis was 0.870 (95%CI 0.797 to 0.925).

**Conclusions:**

The study found that preoperative subjective sleep quality was strongly associated with POD during major non-cardiac surgery. Additionally, PSQI combined with age, BMI, and operation time improved POD prediction.

## Introduction

Postoperative delirium (POD), occurring in the hours to days after surgical procedures, is an acute form of brain dysfunction and it is associated with numerous adverse outcomes, including decreased physical function, declined activities of daily living, prolonged hospitalization, and increased risks of rehospitalization and death [1–4]. Nevertheless, the pathogenesis of POD is unclear, and the efficacy and acceptability of pharmacological interventions for the treatment is uncertain, so identifying associated risk factors are particularly crucial to preventing its occurrence [5–7].

Sleep is a natural state of reduced arousal that promotes the organismal health and cognitive function. Self-reported sleep quality has been associated with cognitive performance [8]. Both short and long sleep may predict cognitive impairment, suggesting a U-shaped association [9]. Poor sleep health is common among perioperative patients [10, 11]. Due to the disease itself, accompanying symptoms, psychological factors and other reasons, patients often suffer from sleep problems [12]. Meanwhile, POD patients may show lethargy or sleep cycle inversion, which may affect the postoperative outcome [13, 14]. It has been verified that sleeping disorders are significantly associated with the increased risk of developing POD [15, 16].

However, whether subjective sleep quality can serve as a predictor for POD in patients undergoing major surgery remains unclear. Thus, the goal of this study was to determine the association and prediction of subjective sleep quality and POD during major non-cardiac surgery.

## Materials and methods

The observational study was conducted in the department of anesthesiology of Ningbo No. 2 Hospital between January 2020 and December 2020. The protocol approved by the Ethics Committee of Ningbo No. 2 Hospital (approval No. PJ-NBEY-KY-2017-029-01), and registered with the Chinese Clinical Trial Registry (www.chictr.org.cn; identifier: ChiCTR-OOC-17,013,414). Before surgery, the patients or their family members provided written informed consent.

### Patient selection

Patients scheduled for non-cardiac major surgery under general anesthesia were eligible for the study. Inclusion criteria: (1) aged 60 years and older; (2) undergoing laparotomy (gastrointestinal tumors surgery) or orthopaedic operation (unilateral hip or knee replacement); (3) American Society of Anesthesiologists (ASA) ≤ grade III. Exclusion criteria: (1) pre-existing neurological or psychiatric conditions (review of medical records); (2) having an alcohol or drug dependency (based on Diagnostic and Statistical Manual of Mental Disorders, fifth edition (DSM-V) criteria); (3) preoperative Mini-Mental State Examination (MMSE) score < 20; (4) Geriatric Depression Scale (GDS) score >5; (5) with impaired vision or hearing, unable to read or speak; (6) surgical duration of greater than 4 h; (7) data not completed or lost to follow-up.

### Sleep quality assessment

Following hospital admission, sleep quality of the patients over the previous month was assessed with the Pittsburgh Sleep Quality Index (PSQI) [17]. The PSQI consists of 19 items categorized into 7 subscales: subjective sleep quality, sleep latency, sleep duration, habitual sleep efficiency, sleep disturbances, use of sleeping medication, and daytime dysfunction. The subscales are scored from 0 to 3 (0, very good; 1, fairly good; 2, fairly bad; and 3, very bad), and the total score ranges from 0 to 21. Higher scores indicate poorer sleep quality. Postoperative sleep quality was assessed by a sleep log, which was revised in accordance with the PSQI.

### Confirmation of POD

The Confusion Assessment Method (CAM) was used by a specially trained staff to diagnose POD [18], that was evaluated twice a day (8:00 am-10:00 am and 4:00 pm-6:00 pm) during the first 7 postoperative days [19]. Meanwhile, the Nursing Delirium Screening Checklist (NU-DESC) was used by trained nurses to screen for POD in patients [20, 21]. In case of a positive screening, the staff used the CAM to diagnose POD again.

### Perioperative management

The patients enrolled in the study underwent general anesthesia with orotracheal intubation. Induced anesthesia was achieved using etomidate, propofol, rocuronium, and sufentanil, then maintain anesthesia was achieved through the use of sevoflurane inhalation, propofol, and remifentanil. Anesthetics were modified to achieve a bispectral index (BIS) between 40 and 60 as a guide to anesthesia depth. Hypotension was defined as a mean arterial pressure (MAP) below 60 mmHg, or 30% of the baseline MAP. Intraoperatively, hypotension was treated with vasoactive drugs immediately and then MAP was maintained at a stable level (20% above or below baseline). In most cases, dexmedetomidine was not restricted, and the anesthesiologist could use it according to the medication’s instructions. As a standard procedure, all patients received postoperative analgesia with a patient-controlled analgesia pump.

### Clinical data collection

Perioperative clinical data were collected as follows: (1) data on age, gender, body mass index (BMI), ASA physical status, years of education and lifestyle habits; (2) preoperative MMSE [22], GDS [23] and PSQI; (3) laboratory tests including Hemoglobin, Albumin, white blood cell count (WBC), and C-reactive protein (CRP); (4) other clinical data including, type of operation, duration of surgery and anesthesia, the use of vasoactive drugs and dexmedetomidine (continuous intravenous infusion during surgery), and estimated blood loss, infusion volume (volume of crystalloids and colloids administered during surgery).

### Statistical analysis

The sample size was estimated using PASS 15.0 software (NCSS, USA). According to the results of previous studies on sleep disorders and POD, the incidence of POD in patients with sleep disorders after non-cardiac surgery was 30-53% [24, 25]. The relative risk (RR) of POD in patients with sleep disorders was 3 times higher than that in patients without sleep disorders. Therefore, this study assumed that the incidence rate of POD in patients with preoperative sleep disorders after non-cardiac surgery was 40%, and the sample size was determined to be 112 cases according to the confidence level of 0.9 and the test level α value of 0.05. The expected postoperative loss of follow-up rate was 10%, and a total of 124 patients needed to be included.

Analysis of the data was performed using SPSS 26.0 statistical analysis software (IBM Corporation, Armonk, NY, USA), and the receiver operating characteristic (ROC) curve was plotted and the area under the ROC (AUROC) value calculated using GraphPad Prism (v8.0.1). Quantitative data were tested for normality firstly. Data with normal distribution were expressed as mean ± standard deviation, while data with non-normal distribution were expressed as median [interquartile range (IQR)], and statistical analysis was conducted by double-tail T-test or U test respectively. Classification data and grade data were expressed as cases or rates (%) and analyzed by Chi-square test or Fisher’s exact test. The relevant confounding factors with *P* value less than 0.1 were screened by univariate analysis, and corrected by multivariate logistic regression. The negative double logarithm likelihood ratio test was used to test the overall importance of the model, and the fit of the model was evaluated by the chi-square test of the Hosmer-Lemeshow fit. All the above results were statistically different when *P* < 0.05 was considered by bilateral test.

## Results

### Patient characteristics

**Fig. 1:**
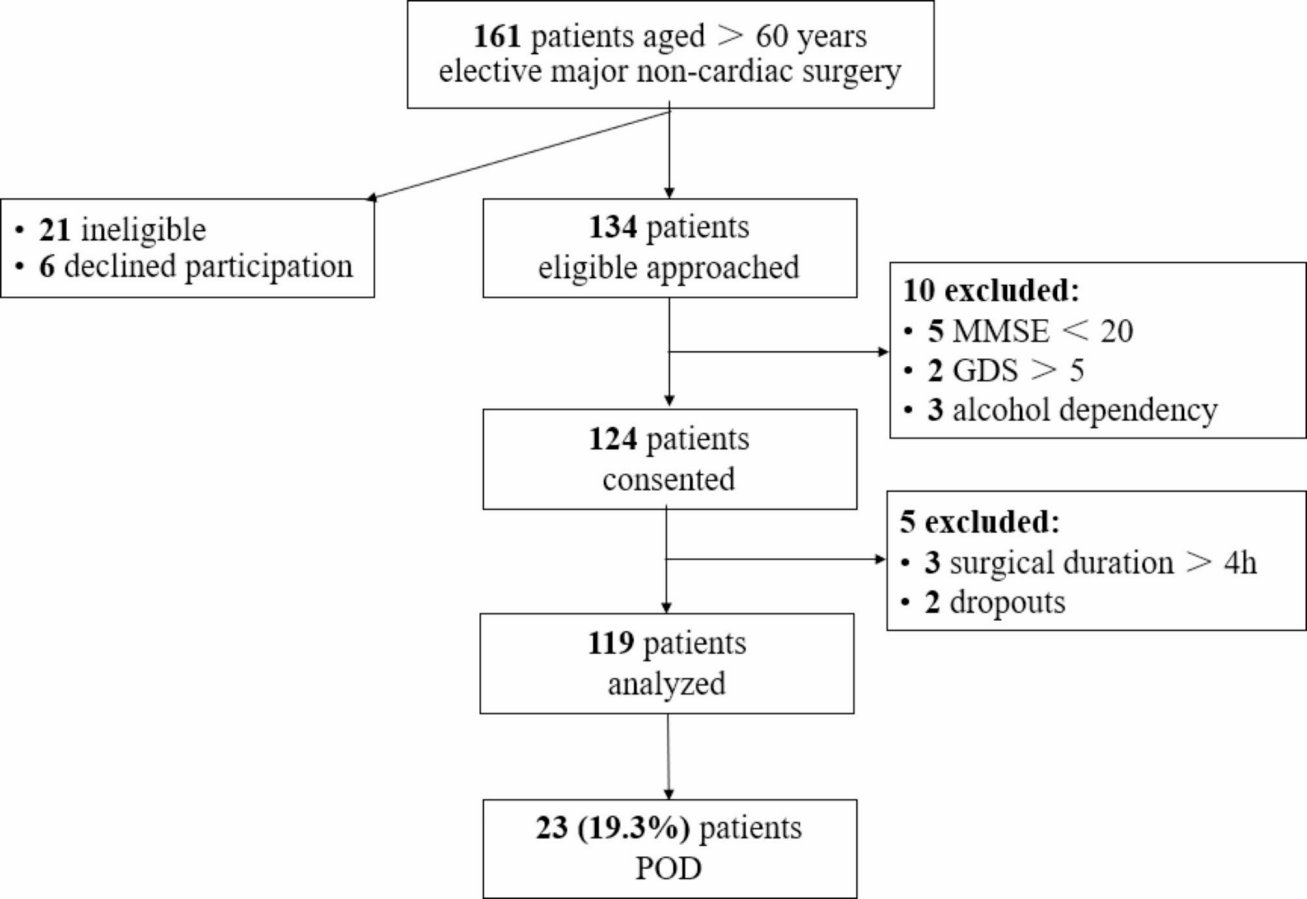
Flow diagram of the study. ***Abbreviations*** *MMSE* Mini-Mental State Examination, *GDS* Geriatric Depression Scale, *POD* Postoperative delirium

Initially, 161 patients aged 60 or older were screened for elective major non-cardiac surgery (Fig. 1). Of these, 21 patients were ineligible, and 6 patients declined to participate. In addition, 15 patients with compound exclusion criteria, and a total of 119 patients were finally followed up. POD occurred in 23 patients, accounting for 19.3%.

The mean age of participants was 71.2 ± 5.5 years, and 56 were males (47.1%). The demographic and baseline characteristics of all the patients are presented in Table 1.

**Table 1:** Demographic and baseline characteristics of participants

**Variable**	**Total**	**POD**	**Without POD**	*P value*
	*n* = 119	*n* = 23	*n* = 96	
Age (year)	71.2 ± 5.5	75.9 ± 7.2	70.0 ± 4.3	0.001 ^*^
Male, *n* (%)	56 (47.1)	11(47.8)	45(46.9)	0.935
BMI (kg/m^2^)	23.6 ± 3.3	21.9 ± 3.2	24.0 ± 3.2	0.005 ^*^
ASA physical status, *n* (%)				0.703
≤II	106 (89.1)	21(91.3)	85(88.5)	
III	13 (10.9)	2(8.7)	11(11.5)	
Education, year (IQR)	6.0 (5.0)	6.0(2.0)	6.0(5.0)	0.177
Constipation, *n* (%)	43 (36.1)	12(52.2)	31(32.3)	0.075
PSQI (point)	7.0 ± 2.4	9.0 ± 2.9	6.7 ± 2.0	0.001 ^*^
Hemoglobin (g/L)	121.6 ± 19.3	118.7 ± 17.4	122.3 ± 19.8	0.416
Albumin (g/L)	40.1 ± 5.5	39.1 ± 5.3	40.3 ± 5.5	0.352
WBC (10^9^/L)	6.3 ± 1.9	6.4 ± 2.1	6.3 ± 1.8	0.914
CRP (mg/L) (IQR)	2.4 (13.3)	4.6(27.7)	2.2(7.4)	0.174

***Abbreviations*** *POD* Postoperative delirium, *BMI* body mass index, *ASA* American Society of Anesthesiologists, *PSQI* Pittsburgh Sleep Quality Index, *WBC* White blood cell count, *CRP* C-reactive protein, *IQR* Interquartile Range

### Correlation analysis between subjective sleep quality and POD

Univariate and multivariate binary logistic regression analysis results for POD were shown in Table 2. Parameters with statistical difference *P* < 0.1 in univariate analysis of POD-related risk factors were selected as variables, that is, age, BMI, constipation, PSQI and operation time were included in multivariate logistic regression analysis. The results showed that the occurrence of POD was closely related to age, BMI, PSQI and operation time (*P* < 0.05). After adjusting for related factors, there was a statistically significant association between PSQI and POD occurrence (OR = 1.422, 95%CI 1.079–1.873, per 1-point increase in PSQI).

**Table 2:** Univariate and multivariate logistic regression analysis of related risk factors for POD

Variable	Univariate analysis			Multivariate analysis	
	*P value*	OR (95%CI)		*P value*	OR (95%CI)
Age (year)	<0.001	1.226 (1.108–1.357)		0.010 ^*^	1.182 (1.042–1.341)
Male (*n)*	0.935	0.963 (0.387–2.394)			
BMI (kg/m^2^)	0.007	0.081 (0.681–0.941)		0.025 ^*^	0.793 (0.647–0.971)
ASA III *(n)*	0.704	0.736 (0.152–3.575)			
Education (year)	0.125	0.911 (0.809–1.026)			
Constipation *(n)*	0.079	2.287 (0.909–5.758)		0.956	0.965 (0.276–3.375)
PSQI (point)	<0.001	1.559 (1.246–1.951)		0.016^*^	1.422 (1.079–1.873)
Hemoglobin (g/L)	0.413	0.991 (0.968–1.013)			
Albumin (g/L)	0.350	0.962 (0.887–1.043)			
WBC (10^9^/L)	0.913	1.013 (0.797–1.288)			
CRP (mg/L)	0.405	1.007 (0.990–1.024)			
Type of operation *(n)*	0.121	2.087 (0.823–5.288)			
Duration of surgery (min)	0.043	1.009 (1.000-1.017)		0.006 ^*^	1.016 (1.005–1.027)
Vasoactive agent *(n)*	0.648	1.236 (0.497–3.075)			
Dexmedetomidine *(n)*	0.458	1.645 (0.442–6.117)			
Bleeding volume (mL)	0.141	0.993 (0.984–1.002)			
Infusion volume (mL)	0.889	1.000 (0.966–1.003)			
Postoperative PSQI (point)	0.508	1.062 (0.889–1.267)			

**Hosmer-Lemesho fit*** P* = 0.161.

***Abbreviations***: *BMI* body mass index, *ASA* American Society of Anesthesiologists, *PSQI* Pittsburgh Sleep Quality Index, *WBC* White blood cell count, *CRP* C-reactive protein. *Type of operation*: laparotomy (gastrointestinal tumors surgery) and orthopaedic operation (unilateral hip or knee replacement)

### Prediction of subjective sleep quality on POD

**Fig. 2:**
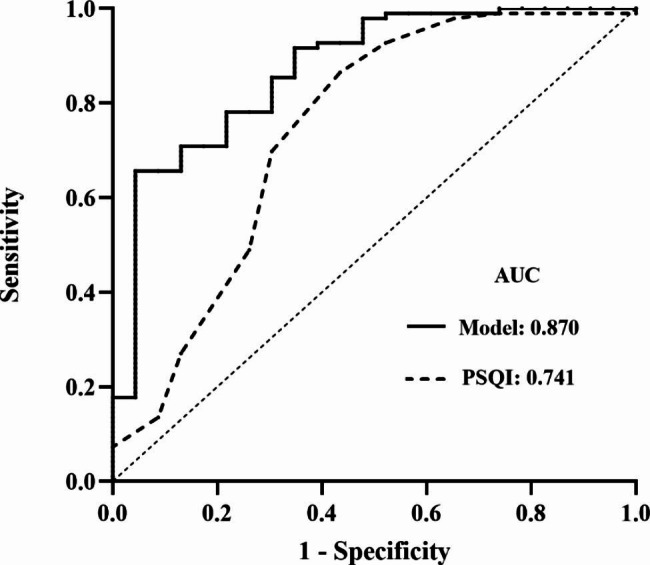
ROC curves of PSQI and the model. ***Abbreviations*** *AUC* area under the ROC curve. *Model* adjusted for age, BMI, constipation, PSQI and operative time

Multivariate logistic regression analysis results were used to develop a prediction model. The ROC curve analysis of PSQI and the model were shown in Fig. 2. It showed that the optimal PSQI cutoff value was 8 based on the Youden index for predicting POD. When using PSQI > 8, the AUROC of PSQI was 0.741 (95%CI 0.635 to 0.817) with a sensitivity and specificity of 56.5% and 86.5%, respectively. The AUROC of the model was 0.870 (95%CI 0.797 to 0.925), corresponding sensitivity and specificity were 97.7% and 65.6%. The results of the Hosmer-Lemeshow goodness-of-fit test (11.785, *P* = 0.161) indicated that the model had a prediction effect.

## Discussion

POD is a great threat and challenge for those patients who undergo major surgery. As seen from previous studies [26–28], it occurs in 5-54% of patients after gastrointestinal surgery and total joint arthroplasty. It is possible that the wide variation in results can be explained by different study populations, as well as partly explained by delirium’s diagnostic approach [29]. The present study used CAM for diagnosis, which is the method used in most studies [30, 31], as well as Nu-DESC was used for screening to improve POD detection rates [32]. As a result of this study, 19.3% (23/119) of participants showed symptoms of POD.

In this study, subjective sleep quality was measured using the PSQI scale, which has become a widely used clinical sleep assessment scale worldwide due to its high reliability and validity [17]. It is indicative of worse sleep quality to have higher PSQI scores. One research indicates that sleep quality is poor if the score is greater than 5 [25]. The study showed that the optimal PSQI cutoff value was 8 based on the Youden index for predicting POD. It is therefore possible to provide better clinical guidance.

In the present study, preoperative subjective sleep quality was significantly associated with an increased risk of developing POD in patients undergoing major non-cardiac surgery. The results of relevant studies support this conclusion [33, 34]. Sleep disturbance was common during perioperative periods, and it adversely affected cognitive function after surgery [14]. We also found that patients had poorer sleep quality after surgery, but this did not seem to predict POD onset. This may be because there were many factors affecting sleep quality after surgery, and we didn’t interfere with them. Furthermore, sleeping disorders may be secondary symptoms of POD, but we did not exclude patients with sleeping disorders diagnosed during or after POD.

Meanwhile, the results showed that age, BMI and operation duration were independent risk factors for POD in our results of univariate analysis. After adjusting for the above factors by multivariate logistic regression, subjective sleep quality was still strongly associated with POD. Then, we developed a predictive model based on the independent risk factors above. The AUC for subjective sleep quality and the model were 0.741 and 0.870, respectively. Clearly, the model is more accurate when predicting POD. However, in clinical applications, sleep quality may be better for intervention due to age and BMI immutability, as well as the uncertainty of the operation time. Therefore, clinicians should pay more attention to evaluating patients’ sleep quality, and seek solutions to improve sleep quality for patients. Those may be beneficial in preventing postoperative delirium after major noncardiac surgery.

It is possible to improve poor sleep quality with non-pharmacological and pharmacological interventions. Sleep disruptions in intensive care units can be reduced through non-pharmacological sleep aids, such as soothing music, earplugs, and eye masks [35]. One meta-analysis revealed that melatonin, which is a hormone produced by the pineal gland, protects against delirium by improving sleep quality and treating inflammation in surgical and intensive care patients [36]. Su et al. demonstrated that the administration of a prophylactic low-dose infusion of dexmedetomidine, an α2 adrenergic receptor agonist, resulted in significantly reduced incidence of POD in post-surgical non-cardiac patients [37].

There were several limitations to this study. First, the study did not employ objective sleep quality tests. The gold standard for measuring sleep is polysomnography (PSG), but it is not applicable due to the particularity of perioperative patients [25]. Moreover, we were seeking a simple way to predict POD. Second, during the hospital stay before surgery, sleep status was not specifically measured. Due to the hospital environment and frequent interruptions, sleep quality in the hospital is usually poor. Third, due to sample size limitations, there is still a residual risk of confusion due to the lack of match with important baseline features in this study. In addition, only patients undergoing specific forms of surgery were included in this study, which was conducted at a single institution in a limited geographical region. Hence, further research on this topic is warranted in order to extend the generalizability of the findings.

## Conclusions

In conclusion, our results revealed that preoperative subjective sleep quality was strongly associated with POD during major non-cardiac surgery. Additionally, PSQI combined with age, BMI and operation time improved POD prediction.

## Data Availability

The datasets used and analysed during the current study are available from the corresponding author on reasonable request.

## Abbreviations

ASA: American society of anesthesiologists
AUROC: Area under the ROC
BIS: Bispectral index
BMI: Body mass index
CAM: Confusion assessment method
CRP: C-reactive protein
GDS: Geriatric depression scale
IQR: Interquartile Range
MMSE: Mini-mental state examination
NU-DESC: Nursing delirium screening checklist
POD: Postoperative delirium
PSQI: Pittsburgh sleep quality index
ROC: Receiver operating characteristic
WBC: White blood cell count
